# Breast-Conserving Therapy Has Better Prognosis for Tumors in the Central and Nipple Portion of Breast Cancer Compared with Mastectomy: A SEER Data-Based Study

**DOI:** 10.3389/fonc.2021.642571

**Published:** 2021-08-12

**Authors:** Jing Wang, Xiaoyu Wang, Zhenyu Zhong, Xue Li, Jiazheng Sun, Jie Li, Jiefeng Huang, Yunhai Li, Guosheng Ren, Hongzhong Li

**Affiliations:** ^1^Department of Endocrine and Breast Surgery, The First Affiliated Hospital of Chongqing Medical University, Chongqing, China; ^2^Chongqing Key Laboratory of Molecular Oncology and Epigenetics, The First Affiliated Hospital of Chongqing Medical University, Chongqing, China; ^3^Department of Ophthalmology, The First Affiliated Hospital of Chongqing Medical University, Chongqing, China; ^4^College of Foreign Languages, Chongqing Medical University, Chongqing, China

**Keywords:** tumors in the central and nipple portion, breast-conserving therapy, mastectomy, prognosis, SEER

## Abstract

**Background and Objectives:**

Currently, the location of primary tumor was an independent prognostic factor of breast cancer. Tumors in the central and nipple portion (TCNP) had poor prognosis compared to other peripheral quadrants. The breast-conserving therapy (BCT) is becoming increasingly common worldwide in breast cancer operations. However, whether the availability of BCT was performed for TCNP remained a matter of debate. We sought to investigate whether BCT was suitable for TCNP with respect to survival outcomes, compared with mastectomy therapy.

**Methods:**

Utilizing the Surveillance, Epidemiology, and End Results (SEER) database, we obtained TCNP breast cancer patients diagnosed during the period of 2010–2015. One-to-one (1:1) propensity score matching (PSM) was applied to construct a matched sample consisting of pairs of BCT and mastectomy groups. Univariate and multivariate Cox proportional hazard models were applied to estimate the factors associated with breast cancer-specific survival (BCSS) and overall survival (OS). Survival analysis was performed with the Kaplan–Meier method.

**Results:**

In the overall cohort, a total of 9,900 patients were enrolled. We found that patients with BCT showed significantly better BCSS (log-rank, *p* < 0.001) and OS (log-rank, *p* < 0.001) than the mastectomy group before PSM. The same finding was also shown in 5,820 patients after PSM. Additionally, none of the subgroups, including age, sex, race, histological grade, AJCC stage, and molecular subtype undergoing mastectomy therapy, had better BCSS than BCT.

**Conclusions:**

Our study was the first research to show that BCT exhibited superior prognosis in the cohort of TCNP from SEER databases than mastectomy therapy. This finding could provide a cue for treatment strategies for suitable TCNP patients, especially those with a strong willingness to conserve their breasts.

## Introduction

As the most common cancer in American women, breast cancer remains second to lung cancer in mortality rate, accounting for about 15% of all cancers this year (1). Although the death rate of female breast cancer has dropped from its peak by 40% since 1989, the decline rate slowed down in recent years (1). Additionally, some early-stage patients still have worse survival in clinical studies (2, 3). A series of studies revealed that age, primary tumor size, tumor location, positive axillary lymph nodes, histological grade, molecular subtype, and BRCA mutation were closely correlated with the prognosis of breast cancer (4–10).

Of note, the location of primary tumor, which has been reported by a wide variety of investigations, is an independent prognostic factor (9, 11–13). A similar finding was demonstrated in subsets of patients with colon, gastric, and lung cancer (14–16). Importantly, tumors in the central and nipple portion (TCNP) had poorer prognosis and more aggressive clinicopathological characteristics than peripheral quadrants (9). With consideration of recurrence rate and survival time, previous surgeons mostly made non-conservative operations for tumor in the central location (17, 18). Recently, with widespread popularization of oncologic concepts and increasingly mature plastic technology, the breast-conserving therapy (BCT), breast-conserving surgery with adjuvant whole breast radiotherapy (RT), is becoming gradually common worldwide (19, 20). Indeed, BCT showed more benefits for patients relative to mastectomy therapy, including advantages such as reducing operative time, diminishing psychological burden, improving cosmetic outcomes, and limiting side effects (21). However, the availability and safety of BCT for TCNP remained a matter of debate (22). Additionally, large-sample studies concerning the comparison of prognosis between BCT and mastectomy therapy for TCNP of breast cancer are scarce.

Therefore, based on the Surveillance, Epidemiology, and End Results (SEER) database, we aimed to explore whether BCT is more suitable for TCNP of breast cancer compared with mastectomy therapy.

## Materials and Methods

### Study Design and Data Sources

**Figure 1:**
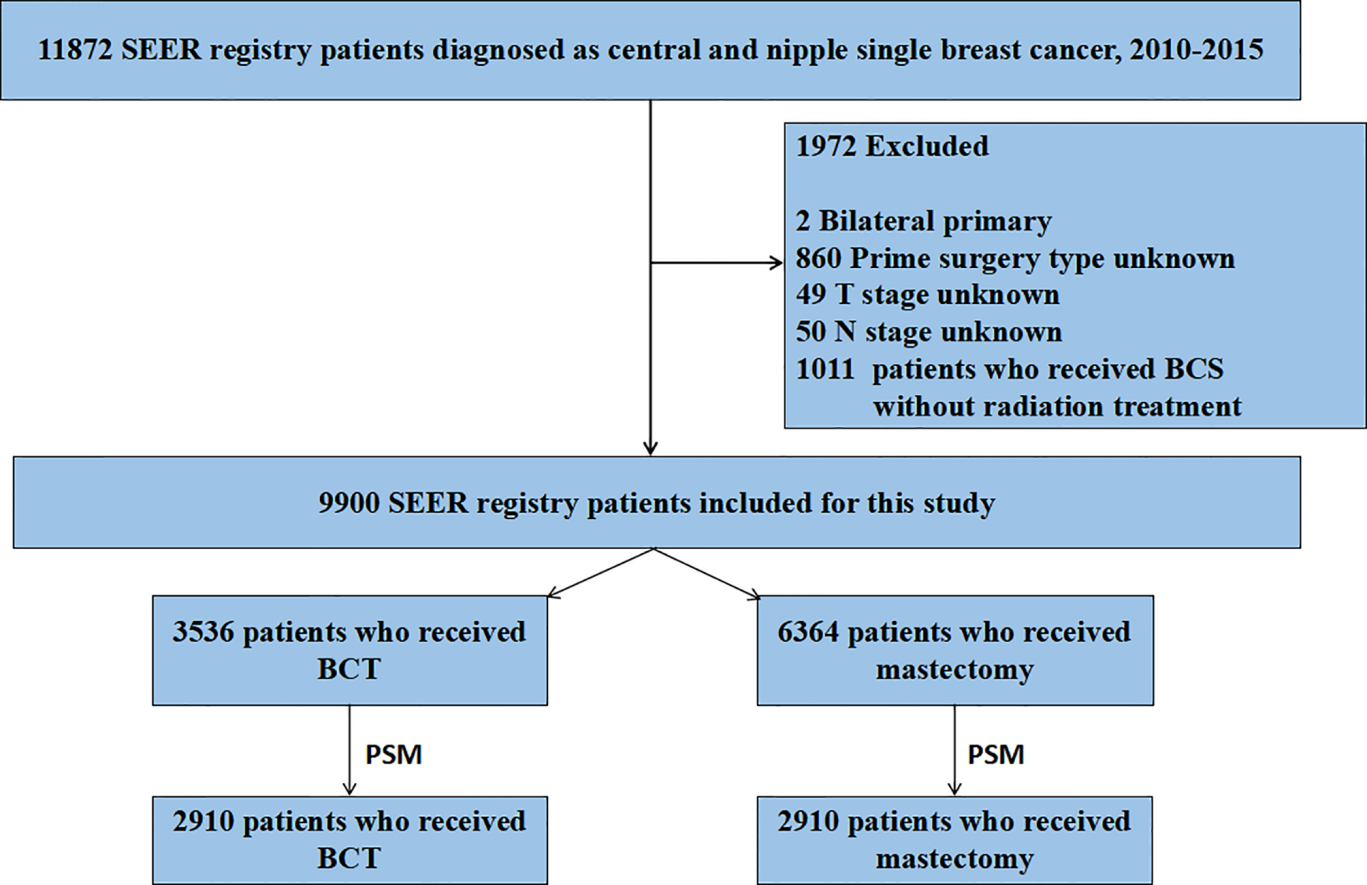
Flow chart for screening patients. SEER, Surveillance Epidemiology, and End Results; PSM, propensity score matching. BCT, breast-conserving therapy.

Using SEER 18 Regs Custom Data (with additional treatment fields), we abstracted Breast Cancer cases from the SEER data released in November 2018. A total of 11,872 patients were originally identified according to the following criteria: tumor in the nipple (C50.0) and central portion (C50.1), the first and only malignant primary tumor, age at diagnosis 20 years, year of diagnosis (2010–2015), race (white, black, Asian/Pacific Islander, and American Indian/Alaska Native), site recode (breast), histological grade (I to IV), AJCC stages (I–IV), breast molecular subtype (Luminal A, Luminal B, HER2 enriched, and Triple Negative), and record of radiation therapy. Criteria for exclusion of the patients were as follows: unknown unilateral tumor (*n* = 2), unknown primary surgery type (*n* = 860), unknown T stage (*n* = 49), unknown N stage (*n* = 50), and treatment of breast-conserving surgery without radiation therapy (*n* = 1,011) (**Figure 1**). A total of 9,900 breast cancer patients were included in this retrospective cohort study.

The primary end points of our study were breast cancer-specific survival (BCSS) and overall survival (OS). BCSS was calculated from the date of diagnosis to the date of death from breast cancer or the last follow-up, and OS was calculated from the date of diagnosis to the date of death for any cause or the last follow-up.

### Statistical Analysis

Statistical analysis was performed by virtue of the SPSS version 21.0 software package (IBM SPSS Statistics, Chicago, IL, USA). The following variables were analyzed: age, sex, race, laterality, histological grade, AJCC stage, tumor size, LN status, distant metastasis, breast molecular subtype, chemotherapy, radiotherapy, and treatment (BCT *vs.* mastectomy). The differences in clinicopathological characteristics between the two groups (BCT and mastectomy therapy) were examined using the Chi-square test. To reduce the obvious differences in baseline covariates and inherent selection bias, we conducted a one-to-one propensity score matching (PSM) analysis (23) between the two treatment groups, including these following variables: age, sex, race, laterality, histological grade, AJCC stage, breast molecular subtype, and chemotherapy. In this PSM, we set the match tolerance as 0.02. The BCSS and OS survival curves were estimated with the Kaplan–Meier method, and survival differences were assessed by the log-rank test. The Cox proportional hazards regression model was conducted on univariate and multivariate analyses of BCSS and OS. Univariate Cox regression analysis was performed for each prognostic variable, and those variables with *p* < 0.05 were included in the multivariate Cox model analysis. All reported *p* values are two-sided, and differences at *p* < 0.05 were considered statistically significant.

## Results

### Demographics and Clinicopathological Findings

This retrospective cohort study included 9,900 patients with breast cancer (9,237 women and 663 men). According to the type of surgery and receipt of RT, all of these patients were divided into two groups: the BCT group (*n* = 3,536, 35.7%) and the mastectomy therapy group (*n* = 6,364, 64.3%). The demographic and clinicopathological characteristics of patients before or after PSM are listed in **Table 1**. Except for laterality, notable differences were detected in relevant variables between the two groups before PSM. The majority of patients in both the BCT group and the mastectomy group were aged older than 50 years (86.6% and 78.2%). We observed that the BCT group, compared with the mastectomy group, exhibited higher rates of female patients (99.3% *vs*. 90.0%, *p* < 0.001), white race (82.4% *vs*. 78.1%, *p* < 0.001), histological grade I and II (27.4% *vs*. 15.7% and 49.5% *vs*. 48.7%, respectively, *p* < 0.001), AJCC stage I (58.7% *vs*. 26.3%), and Luminal A (81.8% *vs*. 73.1%, *p* < 0.001). Besides, the proportion of patients with tumors smaller than 2 cm and negative lymph node (LN) metastasis was also higher in the BCT group than in the mastectomy group (73.1% *vs*. 38.4% and 73.4% *vs*. 48.0%, respectively, *p* < 0.001). Moreover, the BCT group presented a lower percentage of chemotherapy than the mastectomy group (32.9% *vs*. 50.5%, *p* < 0.001). Last but not least, a total of 1,578 breast reconstruction cases of 9,900 patients were in the mastectomy group (**Table 1**).

**Table 1:** Clinicopathological features of the central and nipple breast cancer patients.

Variables	Data before PSM *N* (%)		*p* ^†^	Data after PSM *N* (%)		*p* ^†^
	BCT 3536 (35.7)	Mastectomy 6364 (64.3)		BCT 2910(50)	Mastectomy 2910(50)	
**Age**			<0.001			<0.001
<40	68 (1.9)	350 (5.5)		67 (2.3)	140 (4.8)	
40–49	408 (11.5)	1039 (16.3)		386 (13.3)	482 (16.6)	
50–59	931 (26.3)	1473 (23.1)		813 (27.9)	646 (22.2)	
60–69	1151 (32.6)	1620 (25.5)		885 (30.4)	750 (25.8)	
≥70	978 (27.7)	1882 (29.6)		759 (26.1)	892 (30.7)	
**Sex**			<0.001			0.231
Female	3512 (99.3)	5725 (90.0)		2886 (99.2)	2877 (98.9)	
Male	24 (0.7)	639 (10.0)		24 (0.8)	33 (1.1)	
**Race**			<0.001			0.916
White	2912 (82.4)	4971 (78.1)		2333 (80.2)	2339 (80.4)	
Black	298 (8.4)	632 (9.9)		266 (9.1)	256 (8.8)	
A/PI^a^	305 (8.6)	711 (11.2)		290 (10.0)	297 (10.2)	
AI/AN^b^	21 (0.6)	50 (0.8)		21 (0.7)	18 (0.6)	
**Laterality**			0.132			0.079
Left	1776 (50.2)	3297 (51.8)		1469 (50.5)	1536 (52.8)	
Right	1760 (49.8)	3067 (48.2)		1441 (49.5)	1374 (47.2)	
**Grade**			<0.001			0.301
I	969 (27.4)	999 (15.7)		690 (23.7)	662 (22.7)	
II	1750 (49.5)	3100 (48.7)		1437 (49.4)	1497 (51.4)	
III	811 (22.9)	2242 (35.2)		777 (26.7)	741 (25.5)	
IV	6 (0.2)	23 (0.4)		6 (0.2)	10 (0.3)	
AJCC^c^ Stage			<0.001			0.767
I	2075 (58.7)	1671 (26.3)		1449 (49.8)	1455 (50.0)	
II	1271 (35.9)	2872 (45.1)		1271 (43.7)	1261 (43.3)	
III	173 (4.9)	1618 (25.4)		173 (5.9)	182 (6.3)	
IV	17 (0.5)	203 (3.2)		17 (0.6)	12 (0.4)	
**Tumor Size (cm)**			<0.001			NA
T1 (<2)	2585 (73.1)	2441 (38.4)		NA	NA	NA
T2 (2–5)	833 (23.6)	2631 (41.3)		NA	NA	NA
T3 (>5)	66 (1.9)	735 (11.5)		NA	NA	NA
T4^d^	52 (1.5)	557 (8.8)		NA	NA	NA
**LN status**			<0.001			NA
N0 (negative)	2596 (73.4)	3054 (48.0)		NA	NA	NA
N1 (<3)	829 (23.4)	2134 (33.5)		NA	NA	NA
N2 (4–9)	86 (2.4)	715 (11.2)		NA	NA	NA
N3 (>9)	25 (0.7)	461 (7.2)		NA	NA	NA
**Subtype**			<0.001			<0.001
Luminal A	2891 (81.8)	4650 (73.1)		2291 (78.7)	2260 (77.7)	
Luminal B	308 (8.7)	853 (13.4)		286 (9.8)	338 (11.6)	
HER2 enriched	113 (3.2)	332 (5.2)		112 (3.8)	152 (5.2)	
Triple-negative	224 (6.3)	529 (8.3)		221 (7.6)	160 (5.5)	
**Chemotherapy**			<0.001			1.000
YES	1163 (32.9)	3217 (50.5)		1103 (37.9)	1807 (62.1)	
NO	2373 (67.1)	3147 (49.5)		1103 (37.9)	1807 (62.1)	
**Radiotherapy**			<0.001			NA
YES	3536 (100)	1903 (29.9)		NA	NA	
NO	0 (0)	4461 (70.1)		NA	NA	
**Reconstruction**			<0.001			NA
Tissue^e^	0 (0)	412 (6.5)		NA	NA	
Implant^f^	0 (0)	588 (9.2)		NA	NA	
Combined^g^	0 (0)	211 (3.3)		NA	NA	
Unknown^h^	0 (0)	367 (5.8)		NA	NA	
NO	3536 (100)	4786 (75.2)		NA	NA	

BCT, breast-conserving treatment; NA, not applicable.

a A/PI includes Asian/Pacific Islander;

b AI/AN includes American Indian/Alaskan native;

c AJCC, American Joint Committee on Cancer;

d T4, Tumor of any size with direct extension to the chest wall and/or to the skin (ulceration or skin nodules);

e Tissue, this breast reconstruction is defined as human tissue such as muscle or skin;

f Implant, this breast reconstruction is defined as artificial prostheses;

g Combined, this breast reconstruction is defined as combined tissue and implant;

h Unknown, patients with breast reconstruction but the filling material is unknown;

^†^p < 0.05 was considered statistically significant.

After 1:1 matching, 2,910 patients in the BCT group were matched as compared with 2,910 patients in the mastectomy group. Except for age and molecular subtype, there were no notable differences between the two treatment groups after PSM.

### Survival Analysis for OS and BCSS

**Figure 2:**
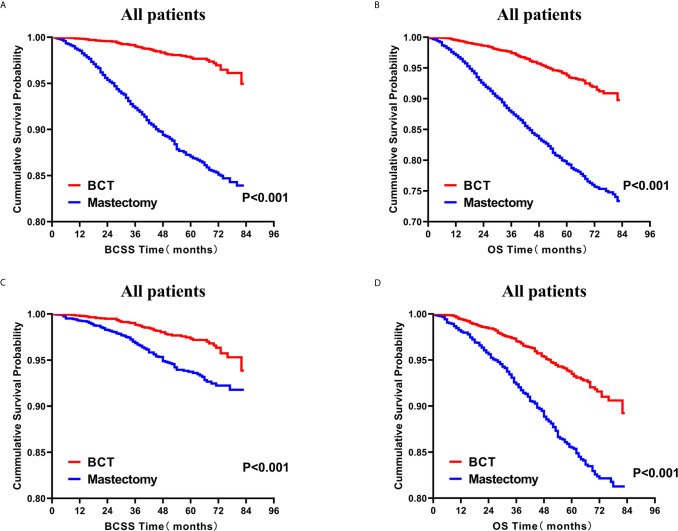
Kaplan–Meier curves for BCSS **(A)** and OS **(B)** by treatment type for all patients before PSM and BCSS **(C)** and OS **(D)** by treatment type for all patients after PSM; BCT versus mastectomy. BCSS, breast cancer specific survival; OS, overall survival; PSM, propensity score matching; BCT, breast-conserving therapy.

**Figure 3:**
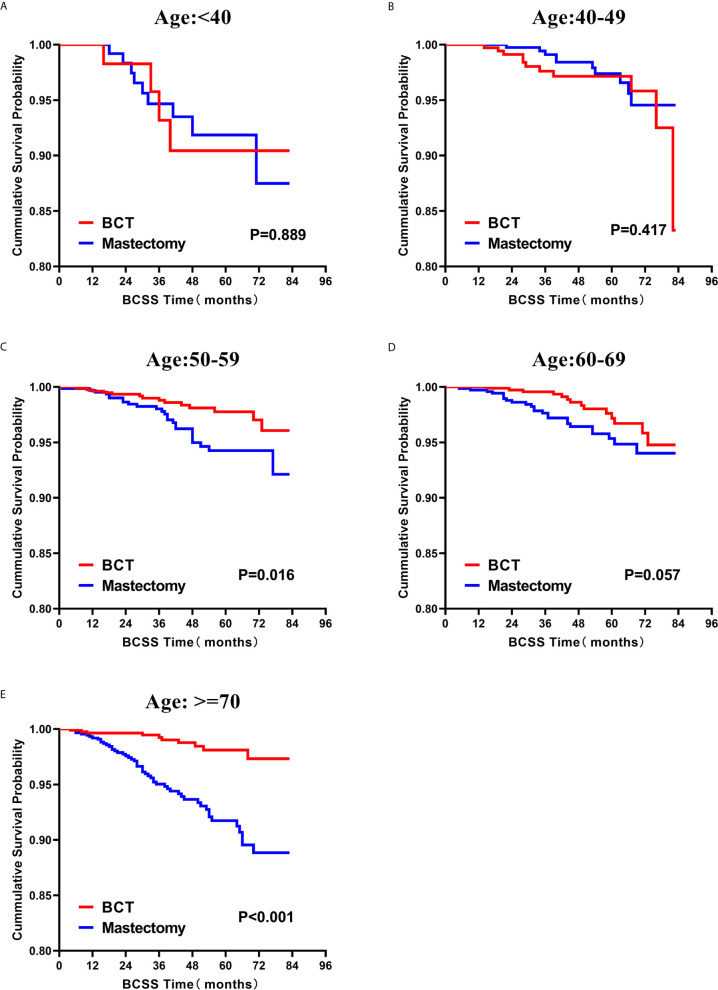
Kaplan–Meier curves for BCSS by treatment type for all patients, stratified by age at diagnosis: **(A)** Age < 40 years. **(B)** Age of 40–49 years. **(C)** Age of 50–59 years. **(D)** Age of 60–69 years. **(E)** Age ≥ 70 years. BCSS, breast cancer specific survival; BCT, breast-conserving therapy.

**Figure 4:**
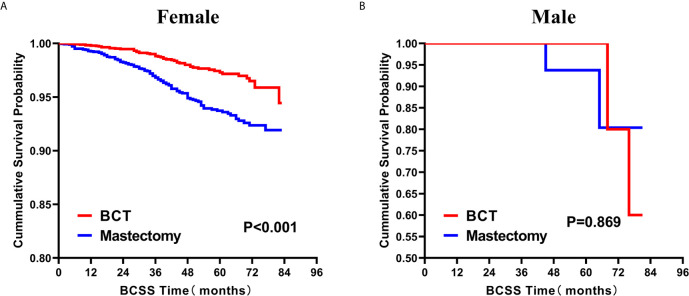
Kaplan–Meier Curves for BCSS by treatment type for all patients, stratified by sex: **(A)** Female. **(B)** Male. BCSS, breast cancer specific survival; BCT, breast-conserving therapy.

**Figure 5:**
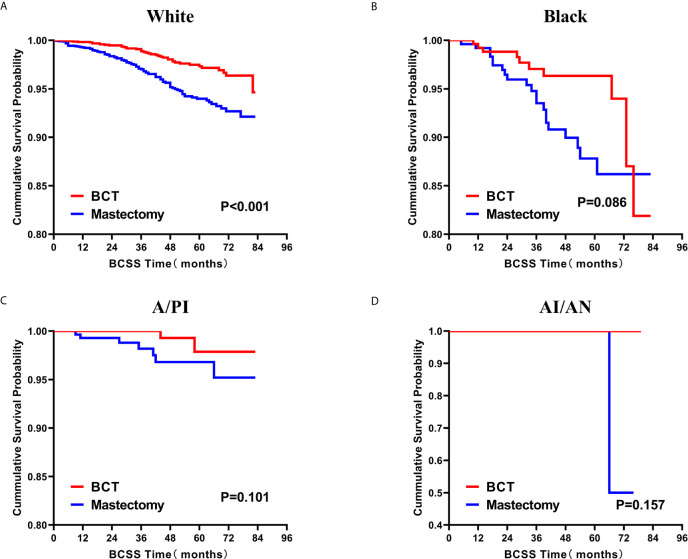
Kaplan–Meier curves for BCSS by treatment type for all patients, stratified by race: **(A)** White. **(B)** Black. **(C)**A/PI. **(D)** AI/AN. BCSS, breast cancer specific survival; BCT, breast-conserving therapy; A/PI, Asian/Pacific Islander; AI/AN, American Indian/Alaskan native.

**Figure 6:**
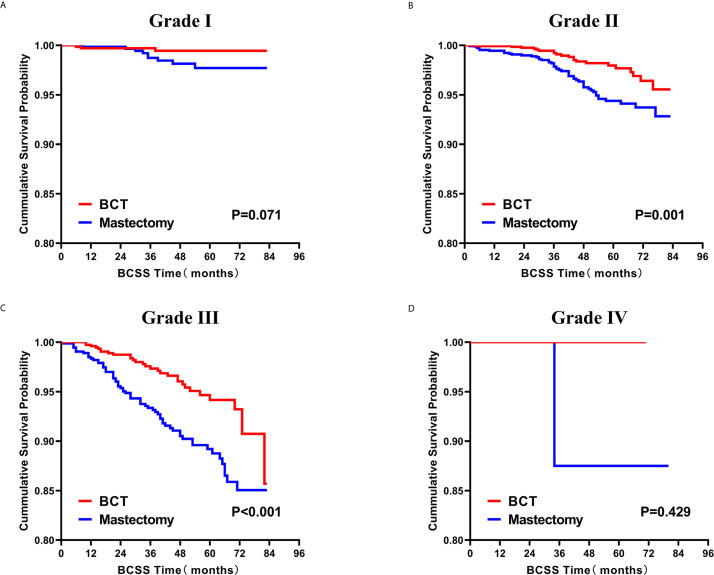
Kaplan–Meier curves for BCSS by treatment type for all patients, stratified by histological grade: **(A)** Grade I.** (B)** Grade II. **(C)** Grade III. **(D)** Grade IV. BCSS, breast cancer specific survival; BCT, breast-conserving therapy.

**Figure 7:**
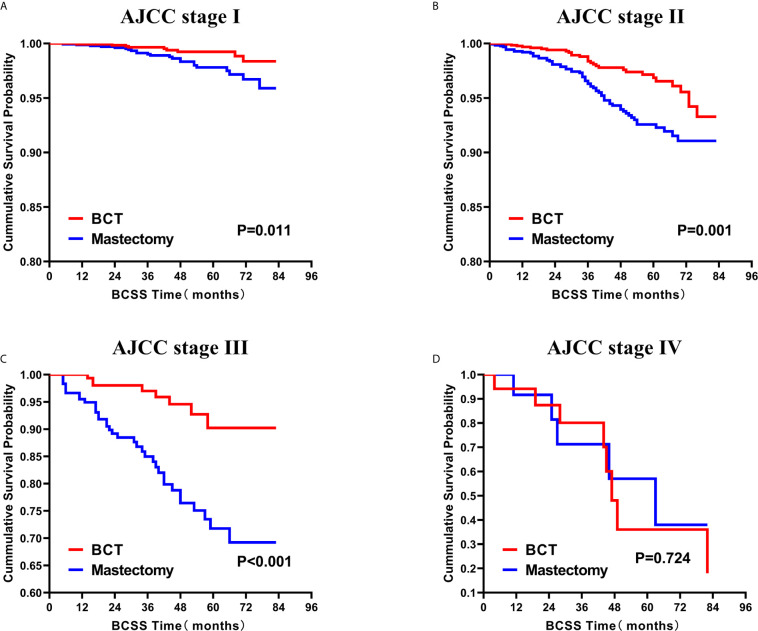
Kaplan–Meier Curves for BCSS by treatment type for all patients, stratified by AJCC stage: **(A)** AJCC stage I.** (B)** AJCC stage II. **(C)** AJCC stage III. **(D)** AJCC stage IV. BCSS, breast cancer specific survival; BCT, breast-conserving therapy.

**Figure 8:**
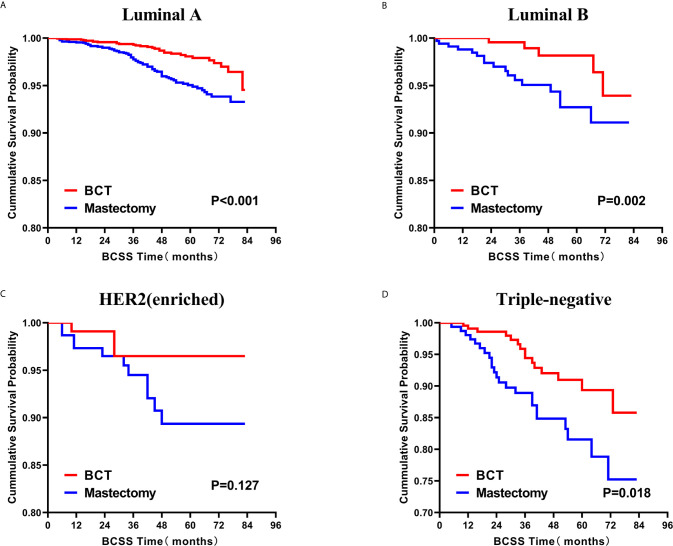
Kaplan–Meier curves for BCSS by treatment type for all patients, stratified by molecular subtype: **(A)** Luminal A. **(B)** Luminal B. **(C)** HER2 enriched. **(D)** Triple-negative. BCSS, breast cancer specific survival; BCT, breast-conserving therapy.

Subsequently, we investigated the outcome of BCSS and OS in patients with TCNP between the BCT group and the mastectomy group, in the use of the Kaplan–Meier method. Before PSM, patients who received BCT showed significantly better BCSS and OS (*p* < 0.001) than those undergoing mastectomy therapy (**Figures 2A, B**). Similarly, we still found the notable difference between the two groups after PSM (*p* < 0.001; **Figures 2C, D**). We next assessed the prognostic values of the BCT group compared with the mastectomy group in various subgroups, including age, sex, race, histological grade, AJCC stage, and molecular subtype (**Figures 3**–**8**, **Supplementary Figures 1–6**). Interestingly, these results showed that the BCT had poorer survival in none of all these clinicopathological parameters subgroups. Compared with the patients who are undergoing mastectomy therapy, BCT was a better prognostic factor for BCSS in patients aged between 50 and 59 or those aged 70 and above (*p* = 0.016 and *p* < 0.001, respectively; **Figures 3C, E**), female patients (*p* < 0.001; **Figure 4A**), those of white race (*p* < 0.001; **Figure 5A**), and those with histological grade II (moderate differentiation) and III (poor differentiation) (*p* = 0.001 and *p* < 0.001, respectively; **Figures 6B, C**). Except for Stage IV, patients with Stage I–III showed better survival with BCT (*p* = 0.011, *p* = 0.001, and *p* < 0.001, respectively; **Figures 7A–C**). Similar outcomes were present in the molecular subtype of patients including Luminal A, Luminal B, and Triple Negative (*p* < 0.001, *p* = 0.002, and *p* = 0.018, respectively; **Figures 8A, B, D**). Besides, as for OS, BCT group still had better prognosis than the mastectomy group in most of the above subgroups (**Supplementary Figures 1**–**6**).

### Prognostic Factors for TCNP Patients

Furthermore, we next explored the prognostic factors associated with BCSS and OS in the cohort of patients. Univariable Cox regression analysis revealed that age, race, histological grade, AJCC stage, molecular subtype, receipt of chemotherapy, and treatment type were important factors associated with BCSS, while age, sex, race, histological grade, AJCC stage, molecular subtype, and treatment type were visibly associated with OS (**Table 2**). After we included the covariates that were clinically worth exploring or had *p* < 0.05 in the univariate analysis into the multivariate analysis, the parameters age, sex, race, histological grade, AJCC stage, molecular subtype, and treatment type were independent prognostic factors of BCSS in TCNP patients. While the factors age, race, histological grade, AJCC stage, molecular subtype, receipt of chemotherapy, and treatment type were significantly associated with OS (**Table 3**).

**Table 2:** Univariable Cox proportional hazard regression model of breast cancer-specific survival (BCSS) and overall survival (OS).

Variable	BCSS		OS	
	HR (95% CI)	*p* ^†^	HR (95% CI)	*p* ^†^
**Age**				
<40	Reference		Reference	
40–49	0.375 (0.188–0.749)	0.005	0.462 (0.237–0.899)	0.023
50–59	0.405 (0.216–0.760)	0.005	0.518 (0.281–0.954)	0.035
60–69	0.376 (0.200–0.706)	0.002	0.797 (0.444–1.431)	0.447
≥70	0.732 (0.405–1.322)	0.301	2.733 (1.566–4.768)	<0.001
**Sex**				
Female	Reference		Reference	
Male	2.254 (0.837–6.073)	0.108	2.096 (1.083–4.056)	0.028
**Race**				
White	Reference		Reference	
Black	2.152 (1.466–3.160)	<0.001	1.463 (1.104–1.939)	0.008
A/PI^a^	0.546 (0.278–1.072)	0.079	0.377 (0.229–0.622)	<0.001
AI/AN^b^	0.983 (0.138–7.031)	0.987	1.894 (0.784–4.579)	0.156
**Laterality**				
Left	Reference		Reference	
Right	0.854 (0.638–1.145)	0.292	0.943 (0.781–1.138)	0.540
**Grade**				
I	Reference		Reference	
II	2.907 (1.579–5.349)	0.001	1.223 (0.935–1.599)	0.142
III	7.192 (3.945–13.111)	<0.001	2.083 (1.586–2.737)	<0.001
IV	7.113 (0.925–54.718)	0.059	1.131 (0.157–8.137)	0.903
AJCC^c^ Stage				
I	Reference		Reference	
II	3.378 (2.271–5.025)	<0.001	1.769 (1.431–2.187)	<0.001
III	12.304 (7.816–19.371)	<0.001	4.389 (3.283–5.867)	<0.001
IV	45.713 (24.019–87.001)	<0.001	12.373 (7.265–21.075)	<0.001
**Breast Subtype**				
Luminal A	Reference		Reference	
Luminal B	1.575 (0.996–2.492)	0.052	1.040 (0.754–1.436)	0.809
HER2 (enriched)	2.355 (1.350–4.110)	0.003	1.686 (1.152–2.466)	0.007
Triple-negative	4.336 (2.996–6.276)	<0.001	2.187 (1.642–2.914)	<0.001
**Chemotherapy**				
YES	Reference		Reference	
NO	0.509 (0.380–0.681)	<0.001	1.317 (1.077–1.610)	0.007
**Treatment Type**				
BCT	Reference		Reference	
Mastectomy	2.319 (1.687–3.188)	<0.001	2.366 (1.926–2.907)	<0.001

BCT, breast-conserving treatment;

a A/PI includes Asian/Pacific Islander;

b AI/AN includes American Indian/Alaskan native;

c AJCC, American Joint Committee on Cancer;

^†^p < 0.05 was considered statistically significant.

**Table 3:** Multivariable Cox proportional hazard regression model of breast cancer-specific survival (BCSS) and overall survival (OS).

Variable	BCSS		OS	
	HR (95% CI)	*p* ^†^	HR (95% CI)	*p* ^†^
**Age**				
<40	Reference	NA	Reference	NA
40–49	0.451 (0.225–0.905)	0.025	0.534 (0.274–1.041)	0.066
50–59	0.617 (0.328–1.164)	0.136	0.669 (0.362–1.236)	0.199
60–69	0. 621 (0.326–1.182)	0.147	1.034 (0.572–1.869)	0.912
≥70	1.048 (0.556–1.976)	0.884	3.029 (1.705–5.380)	<0.001
**Sex**				
Female	Reference	NA	Reference	NA
Male	2.888 (1.062–7.854)	0.038	2.429 (1.249–4.724)	0.009
**Race**				
White	Reference	NA	Reference	NA
Black	1.831 (1.237–2.708)	0.002	1.574 (1.184–2.093)	0.002
A/PI^a^	0.648 (0.328–1.277)	0.210	0.458 (0.277–0.757)	0.002
AI/AN^b^	1.460 (0.203–10.524)	0.707	3.038 (1.248–7.392)	0.014
**Grade**				
I	Reference	NA	Reference	NA
II	1.984 (1.069–3.682)	0.030	1.009 (0.768–1.327)	0.947
III	3.474 (1.834–6.581)	<0.001	1.553 (1.148–2.101)	0.004
IV	3.813 (0.488–29.816)	0.202	0.573 (0.079–4.165)	0.582
AJCC^c^ Stage				
I	Reference	NA	Reference	NA
II	3.167 (2.097–4.785)	<0.001	1.845 (1.479–2.301)	<0.001
III	10.434 (6.435–16.916)	<0.001	4.577 (3.359–6.235)	<0.001
IV	43.105 (21.902–84.834)	<0.001	11.997 (6.934–20.756)	<0.001
**Breast Subtype**				
Luminal A	Reference	NA	Reference	NA
Luminal B	1.050 (0.648–1.700)	0.844	1.082 (0.772–1.515)	0.649
HER2 (enriched)	1.531 (0.849–2.762)	0.157	1.558 (1.038–2.338)	0.032
Triple-negative	3.325 (2.190–5.049)	<0.001	2.176 (1.594–2.971)	<0.001
**Chemotherapy**				
YES	Reference	NA	Reference	NA
NO	1.084 (0.749–1.570)	0.668	1.374 (1.072–1.762)	0.012
**Treatment Type**				
BCT	Reference	NA	Reference	NA
Mastectomy	2.359 (1.709–3.258)	<0.001	2.197 (1.785–2.705)	<0.001

BCT, breast-conserving treatment; NA, not applicable.

a A/PI includes Asian/Pacific Islander;

b AI/AN includes American Indian/Alaskan native;

c AJCC, American Joint Committee on Cancer;

^†^p < 0.05 was considered statistically significant.

## Discussion

To our knowledge, this is the first research to explore the role of different treatments on survival of TCNP breast cancer patients. In the retrospective analysis of 9,900 cases, we uncovered that the BCT group exhibited better BCSS and OS than mastectomy group. Furthermore, we also conducted a subgroup analysis of all the independent influencing parameters for BCSS, including age, sex, race, histological grade, AJCC stage, and molecular subtype. We observed that none of subgroups undergoing the mastectomy therapy had better BCSS than BCT.

National Comprehensive Cancer Network (NCCN) guidelines and relevant studies demonstrated that the long-term survival rate of the BCT is similar to that of the mastectomy therapy. Therefore, BCT should be recommended for patients with early breast cancer (24–27). A Dutch study showed that BCT, compared with mastectomy therapy, was significantly correlated with improved 10-year overall survival in the whole cohort of 37,207 T_1-2_N_0-1_M_0_ stage patients (28). Additionally, another population-based study on 10-year follow-up for 3,071 T_1-2_N_2_ stage breast cancer showed that patients who underwent BCT had superior survival time and lower rate of distant metastasis and regional recurrence (29). Our same findings further confirmed the availability and security of BCT and the range of application, such as AJCC stage I–III. These findings also agreed with the NCCN guidelines and Chinese breast cancer guidelines that patients in stage III (except for inflammatory breast cancer) who have been downgraded by neoadjuvant chemotherapy to meet the criteria for breast preservation can also be carefully considered (22, 24). Therefore, BCT has been gradually accepted by more surgeons and popularized by more patients worldwide in recent years. However, which type of patients undergoing BCT would have longer survival time and better quality of life is still an important unsolved issue.

Indeed, tumor location has been proven to be a strong predictive factor for survival (9). However, the opinion of whether BCT is suitable for TCNP breast cancer patients remains controversial (30–32). A study of 105,037 patients showed that TCNP was significantly correlated with older years, larger tumor sizes, higher AJCC stage, and worse survival time relative to other peripheral quadrants (9). Additionally, Chinese breast cancer guidelines indicated that tumor in the nipple was one of the relative contraindications for BCT (22). Intriguingly, our research of 9,900 AJCC stage I–IV TCNP patients showed that BCT exhibited better prognosis in most clinicopathological subgroups, supporting another recent investigation on early-stage T_1_ or T_2_ centrally located breast cancer (32). Our results constituting a wide range and a variety of influential factors from the SEER database provided more convincing evidence that BCT may not be a relative contraindication in clinical decision-making. Additionally, we consider that the superior BCSS outcome of BCT, to some extent, may due to the role of RT. Previous studies had demonstrated that timely adjuvant RT after BCT could decrease locoregional recurrence and distant metastasis (33–35). Another pre-clinical study also demonstrated that RT could potentially induce an anti-tumoral immune response in breast cancer (36).

Currently, breast cancers are classified into four distinct subtypes: Luminal A, Luminal B, HER2-enriched, and TNBC, according to the presence or absence of molecular markers for estrogen or progesterone receptors and HER2 (8). These subtypes have distinct risk profiles and treatment strategies (37). In particular, TNBC tends to exhibit a more aggressive clinical biological characteristics and worse prognosis than other subtypes (8, 38, 39). However, several professionals indicated that BCT might not be considered a contraindication for TNBC (40, 41). From our analysis of the TNBC subgroup, although the BCT group had a higher proportion of TNBC, it showed a superior BCSS survival than mastectomy therapy.

Of note, the application of BCT for TCNP patients should allow for an adequate safety tumor-free margin of at least 2 mm (22). This undoubtedly increases technical difficulties in operations and makes surgeons hesitate to conduct BCT in consideration of locoregional recurrence (42). However, oncoplastic breast surgery (OBS) could broaden the general indication of BCT by achieving wider excision margins, which could ensure better local control of the disease (43). Eggemann, Holm et al. had provided excellent applicability of the B technique for breast cancer localized in the central portion of the breast (20). An inverted T method invented by McCulley et al. also made a similar satisfactory outcome for TCNP (44). Additionally, when BCT was implemented with the OBS technique, the rate of cosmetic failure could drop to <7% within 2 years and <10% within 5 years (45). Kijima et al. produced good cosmetic results for TCNP by multifarious flaps, including Latissimus dorsi mini flap, Grisotti flap, hole-shaped skin glandular flap, and Free dermal fat graft (19). Therefore, surgeons could consider BCT for more TCNP patients, with the help of the OBS technique.

This present study has several potential limitations. Firstly, we could not obtain information about disease recurrence from the SEER database. Therefore, we could not compare the different rates of locoregional recurrence between BCT and mastectomy therapy. Secondly, SEER could not provide information about neoadjuvant therapy. Some patients in the BCT group should undergo neoadjuvant chemotherapy before operation to meet the indication of breast conserving. Therefore, we could not assess the potential role of neoadjuvant therapy on our clinical outcomes. Thirdly, the SEER database collected large numbers of patients’ information from 18 population-based cancer registries. Some clinicopathologic information may be miscoded or missed during the registration process. For instance, patients’ BRCA gene mutations, Ki-67 in immunohistochemical analysis, were not provided in the current dataset.

Collectively, our study was the first investigation to show that BCT exhibited better prognosis than mastectomy therapy in the cohort of TCNP from SEER databases. This finding could provide a cue for treatment strategies for suitable TCNP patients, especially those with a strong willingness to conserve their breasts. However, some other factors, such as the potential locoregional recurrence rate, and whether physical condition could withstand the adverse effects of radiotherapy, should also be taken into account. Therefore, whole-process therapeutic strategies for TCNP patients still warrant further in-depth research.

## Data Availability Statement

The datasets presented in this study can be found in online repositories. The names of the repository/repositories and accession number(s) can be found in the article/**Supplementary Material**.

## Author Contributions

Conception and design: JW, HL, and GR. Data acquisition, analysis, and interpretation: JW, ZZ, and XL. Material support: XL, XW, YL, and HL. Study supervision: GR. All authors contributed to the article and approved the submitted version.

## Funding

This study was supported by the National Natural Science Foundation of China (#31420103915 and #81472475).

## Conflict of Interest

The authors declare that the research was conducted in the absence of any commercial or financial relationships that could be construed as a potential conflict of interest.

## Publisher’s Note

All claims expressed in this article are solely those of the authors and do not necessarily represent those of their affiliated organizations, or those of the publisher, the editors and the reviewers. Any product that may be evaluated in this article, or claim that may be made by its manufacturer, is not guaranteed or endorsed by the publisher.

## Supplementary Material

Supplementary Figure 1 Kaplan-Meier Curves for OS by Treatment Type for All Patients, Stratified by Age at Diagnosis: **(A)** Age <40 years. **(B)** Age of 40-49 years. **(C)** Age of 50-59 years. **(D)** Age of 60-69 years. **(E)** Age ≥ 70 years. BCSS, breast cancer specific survival; BCT, breast-conserving therapy.

Supplementary Figure 2 Kaplan-Meier Curves for OS by Treatment Type for All Patients, Stratified by Sex: **(A)** Female. **(B)** Male. BCSS, breast cancer specific survival; BCT, breast-conserving therapy.

Supplementary Figure 3 Kaplan-Meier Curves for OS by Treatment Type for All Patients, Stratified by Race: **(A)** White. **(B)** Black. **(C)** A/PI. **(D)** AI/AN. BCSS, breast cancer specific survival; BCT, breast-conserving therapy; A/PI, Asian/Pacific Islander; AI/AN, American Indian/Alaskan native.

Supplementary Figure 4 Kaplan-Meier Curves for OS by Treatment Type for All Patients, Stratified by Histological Grade: **(A)** Grade I. **(B)** Grade II. **(C)** Grade III. **(D)** Grade IV. BCSS, breast cancer specific survival; BCT, breast-conserving therapy.

Supplementary Figure 5 Kaplan-Meier Curves for OS by Treatment Type for All Patients, Stratified by AJCC stage: **(A)** AJCC stage I. **(B)** AJCC stage II. **(C)** AJCC stage III. **(D)** AJCC stage IV. BCSS, breast cancer specific survival; BCT, breast-conserving therapy.

Supplementary Figure 6 Kaplan-Meier Curves for OS by Treatment Type for All Patients, Stratified by Molecular Subtype: **(A)** Luminal A. **(B)** Luminal B. **(C)** HER2 enriched. **(D)** Triple-negative. BCSS, breast cancer specific survival; BCT, breast-conserving therapy.
