# Supplementary figures and images for: Breast-Conserving Therapy Has Better Prognosis for Tumors in the Central and Nipple Portion of Breast Cancer Compared with Mastectomy: A SEER Data-Based Study
Source: Front Oncol. 2021 Aug 12;11:642571. doi: 10.3389/fonc.2021.642571 (PMC8397465; doi:10.3389/fonc.2021.642571)

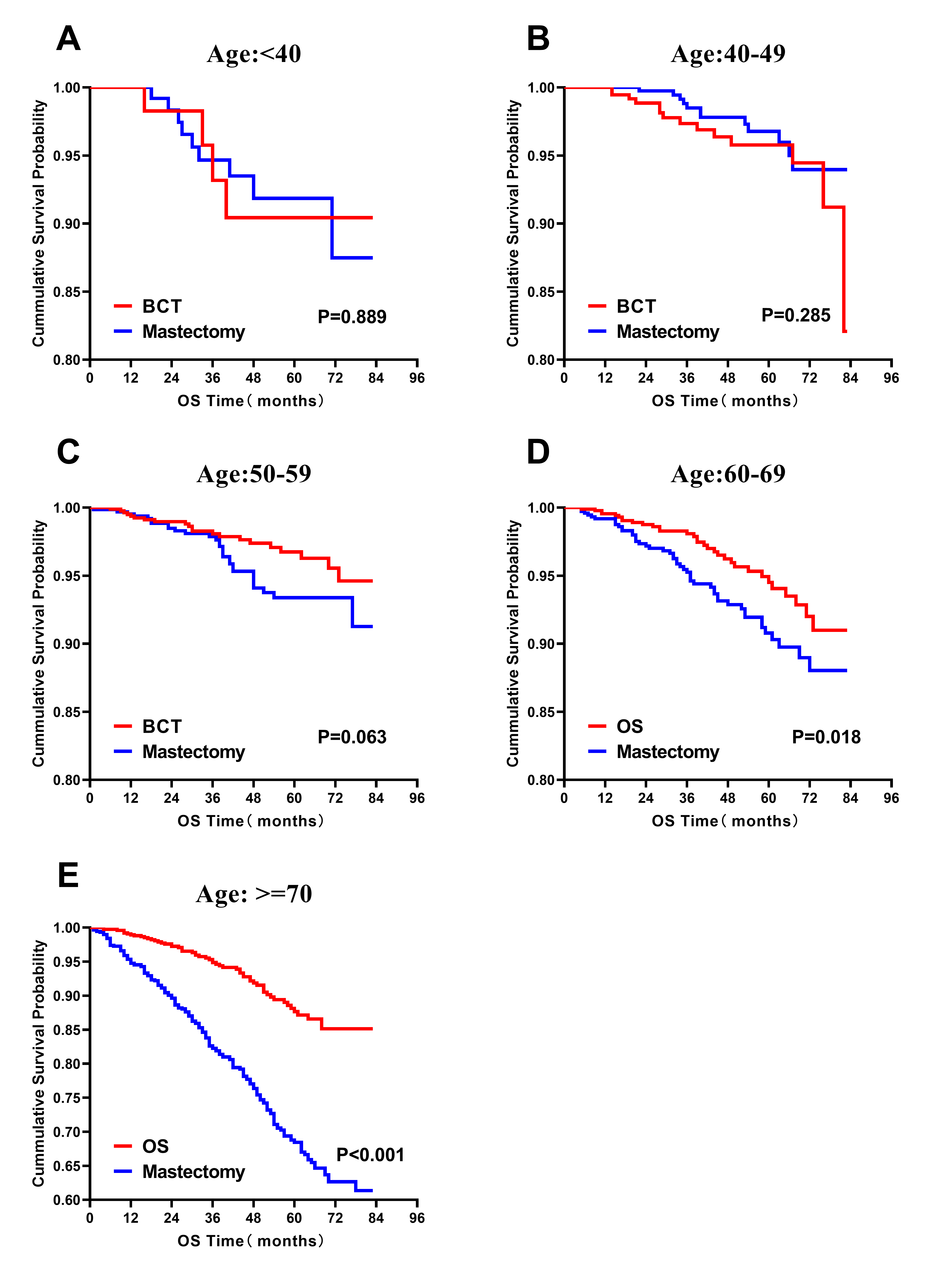

Supplement: Supplementary Figure 1 — Kaplan-Meier Curves for OS by Treatment Type for All Patients, Stratified by Age at Diagnosis: (A) Age <40 years. (B) Age of 40-49 years. (C) Age of 50-59 years. (D) Age of 60-69 years. (E) Age ≥ 70 years. BCSS, breast cancer specific survival; BCT, breast-conserving therapy. [file Image_1.tif]

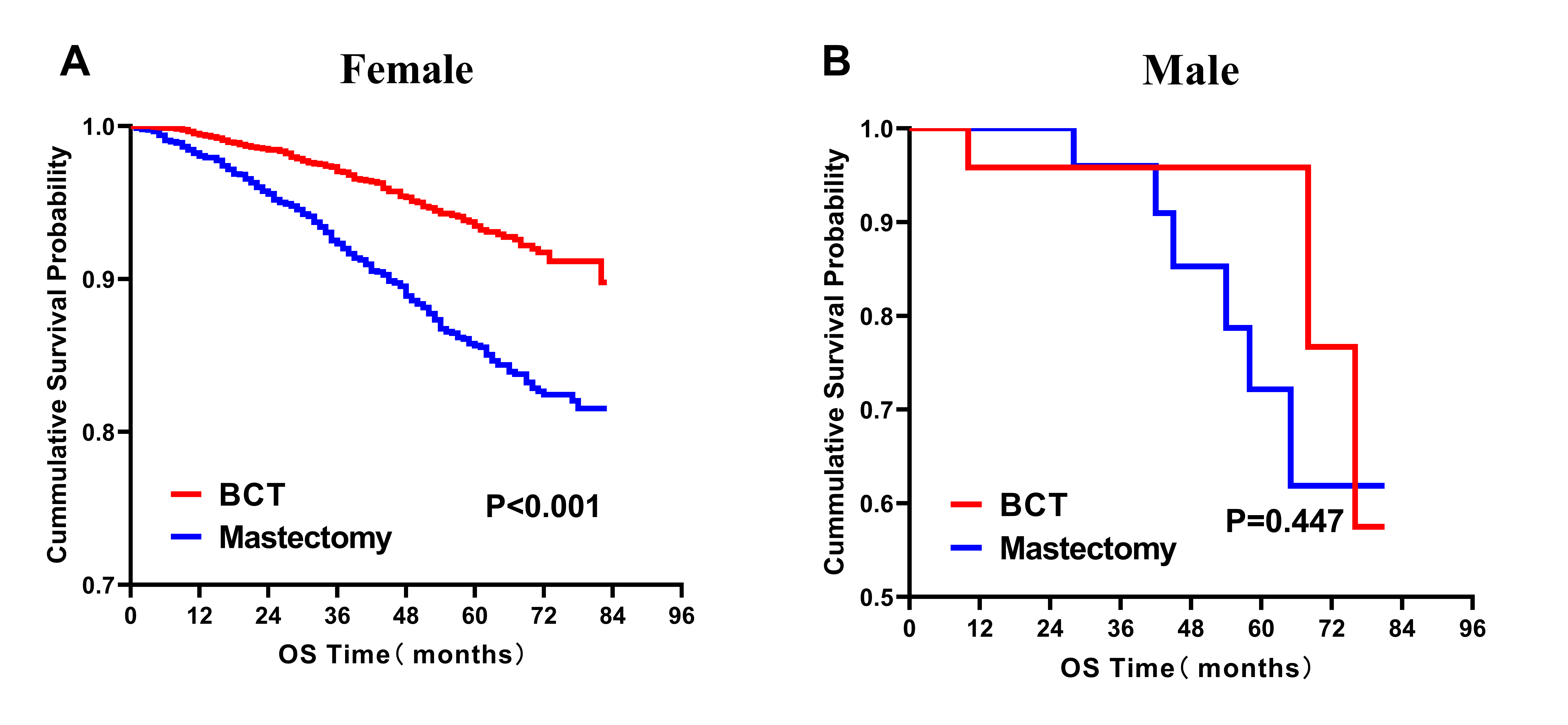

Supplement: Supplementary Figure 2 — Kaplan-Meier Curves for OS by Treatment Type for All Patients, Stratified by Sex: (A) Female. (B) Male. BCSS, breast cancer specific survival; BCT, breast-conserving therapy. [file Image_2.tif]

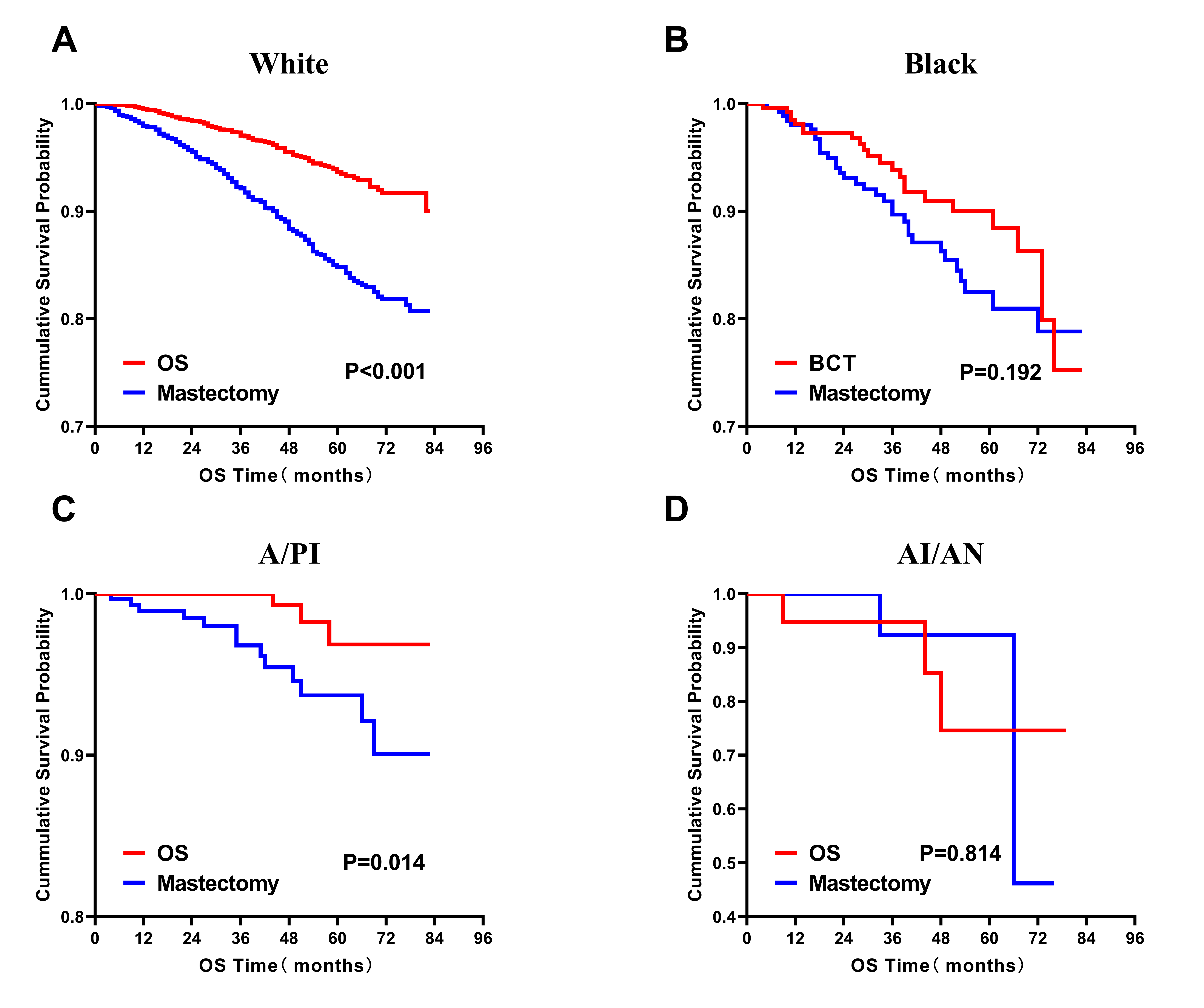

Supplement: Supplementary Figure 3 — Kaplan-Meier Curves for OS by Treatment Type for All Patients, Stratified by Race: (A) White. (B) Black. (C) A/PI. (D) AI/AN. BCSS, breast cancer specific survival; BCT, breast-conserving therapy; A/PI, Asian/Pacific Islander; AI/AN, American Indian/Alaskan native. [file Image_3.tif]

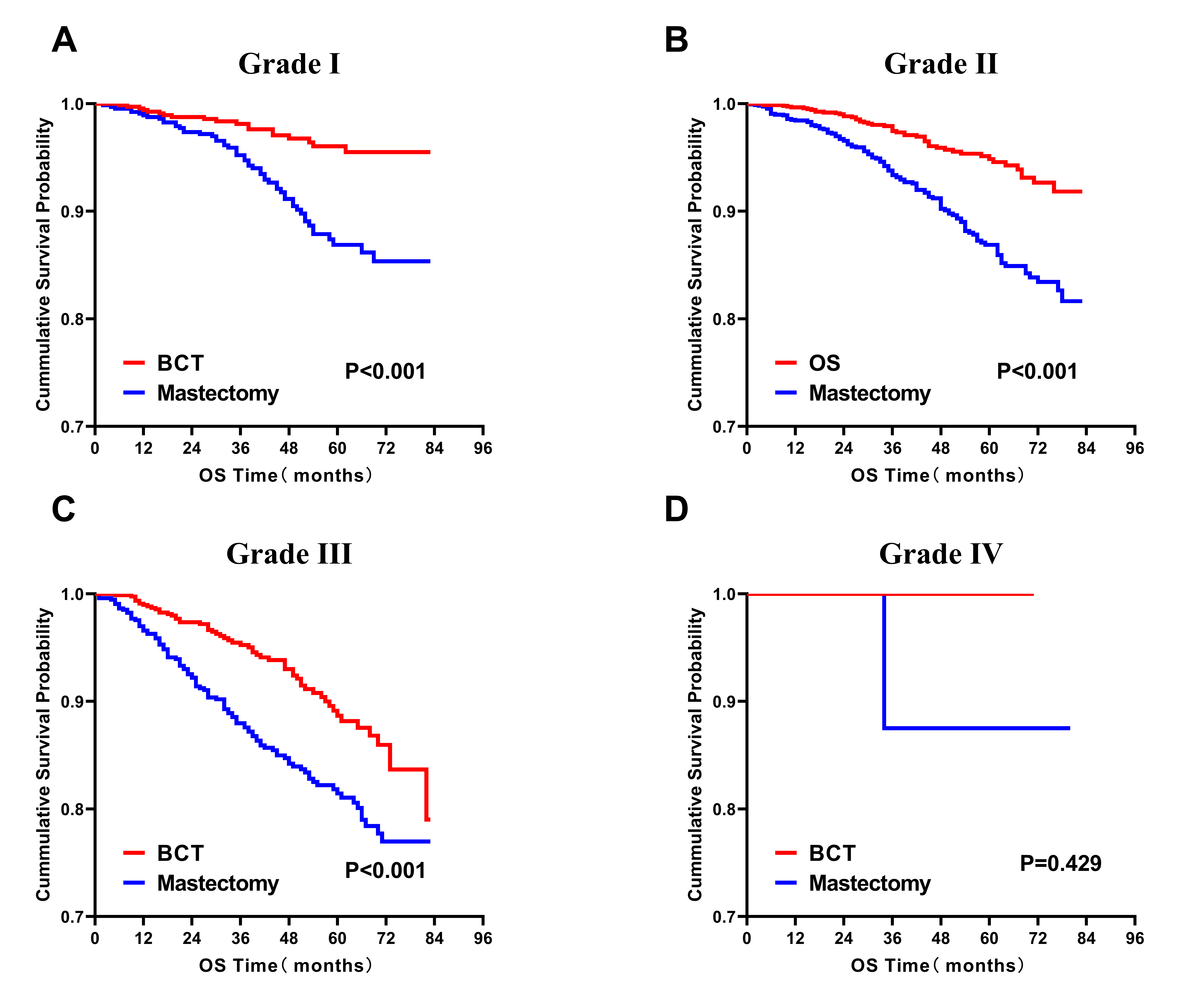

Supplement: Supplementary Figure 4 — Kaplan-Meier Curves for OS by Treatment Type for All Patients, Stratified by Histological Grade: (A) Grade I. (B) Grade II. (C) Grade III. (D) Grade IV. BCSS, breast cancer specific survival; BCT, breast-conserving therapy. [file Image_4.tif]

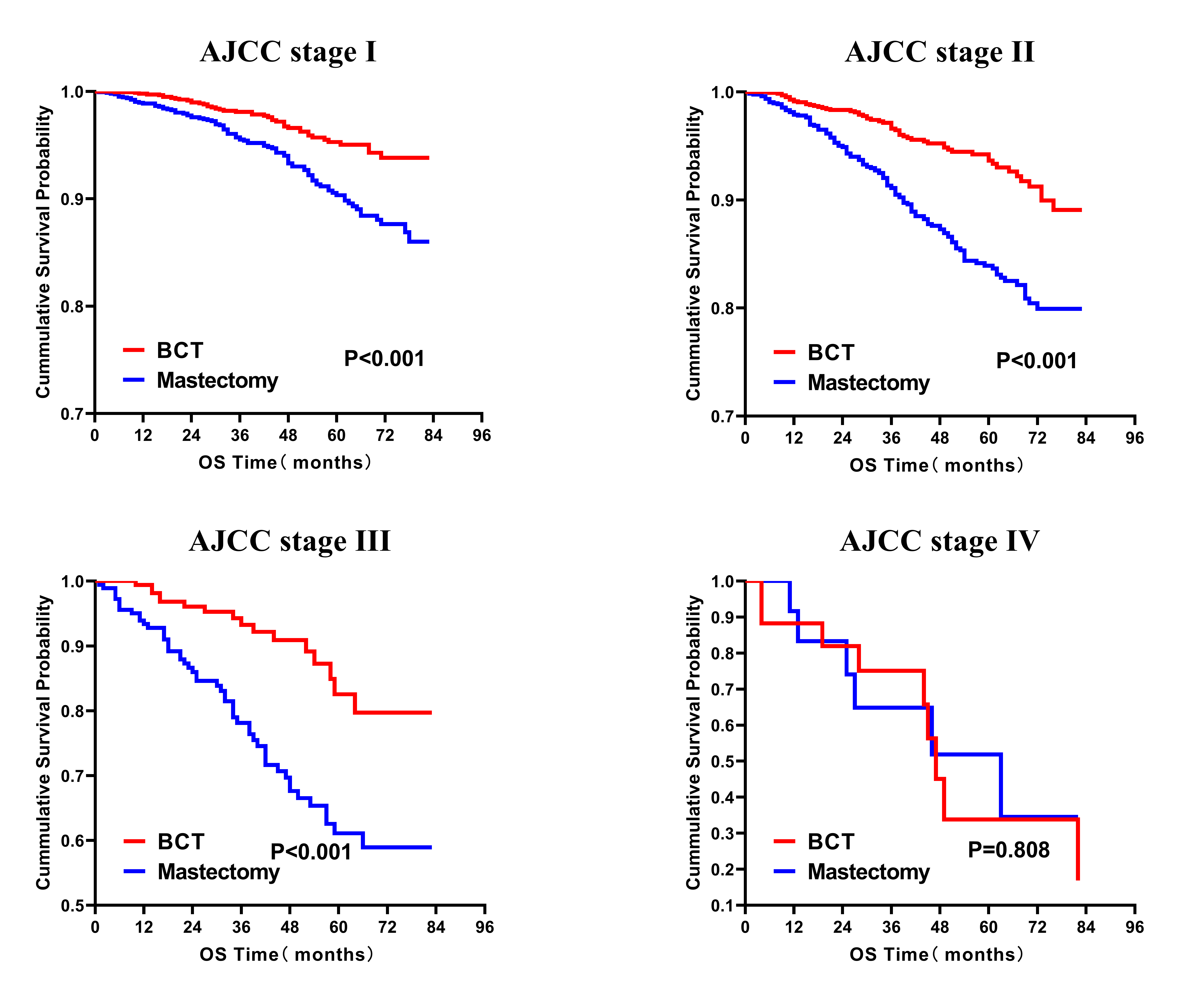

Supplement: Supplementary Figure 5 — Kaplan-Meier Curves for OS by Treatment Type for All Patients, Stratified by AJCC stage: (A) AJCC stage I. (B) AJCC stage II. (C) AJCC stage III. (D) AJCC stage IV. BCSS, breast cancer specific survival; BCT, breast-conserving therapy. [file Image_5.tif]

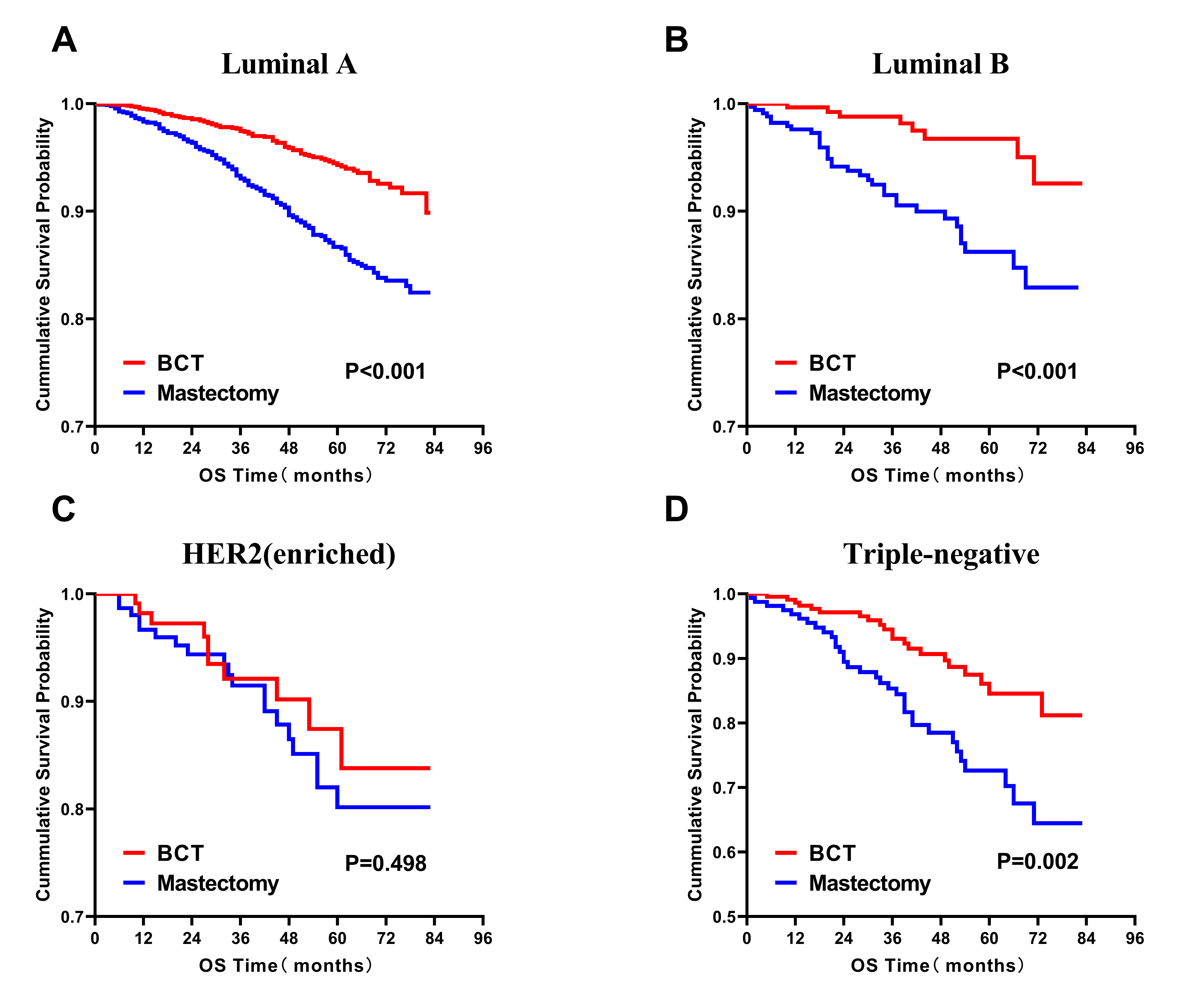

Supplement: Supplementary Figure 6 — Kaplan-Meier Curves for OS by Treatment Type for All Patients, Stratified by Molecular Subtype: (A) Luminal A. (B) Luminal B. (C) HER2 enriched. (D) Triple-negative. BCSS, breast cancer specific survival; BCT, breast-conserving therapy. [file Image_6.tif]
